# The Effect of Co-infection of Food-Borne Pathogenic Bacteria on the Progression of *Campylobacter jejuni* Infection in Mice

**DOI:** 10.3389/fmicb.2018.01977

**Published:** 2018-08-22

**Authors:** Gang Wang, Yufeng He, Xing Jin, Yonghua Zhou, Xiaohua Chen, Jianxin Zhao, Hao Zhang, Wei Chen

**Affiliations:** ^1^State Key Laboratory of Food Science and Technology, Jiangnan University, Wuxi, China; ^2^School of Food Science and Technology, Jiangnan University, Wuxi, China; ^3^Key Laboratory of National Health and Family Planning Commission on Parasitic Disease Control and Prevention, Jiangsu Provincial Key Laboratory on Parasite and Vector Control Technology, Jiangsu Institute of Parasitic Diseases, Wuxi, China; ^4^College of Life Sciences and Environment, Hengyang Normal University, Hengyang, China; ^5^International Joint Research Laboratory for Probiotics, Jiangnan University, Wuxi, China; ^6^Institute of Food Biotechnology, Jiangnan University, Yangzhou, China; ^7^National Engineering Research Center for Functional Food, Jiangnan University, Wuxi, China; ^8^Beijing Innovation Centre of Food Nutrition and Human Health, Beijing Technology and Business University, Beijing, China

**Keywords:** *Campylobacter*, food-borne pathogenic bacteria, co-infection, campylobacteriosis, gut microbiota, butyric acid

## Abstract

*Campylobacter* is a well-known food-borne pathogen that causes human gastroenteritis. Food products that contain *Campylobacter* may also be contaminated by other pathogens, however, whether this multiple contamination leads to more severe infection remains unclear. In this study, mice were gavaged with *Campylobacter jejuni* and other food-borne pathogenic bacteria to mimic a multiple infection. It was demonstrated that the *C. jejuni* load was elevated when the mice were co-infected with *C. jejuni* and *Salmonella typhimurium*, and the campylobacteriosis that followed was also enhanced, with features of decreased body weight, heavier bloody stools and more pronounced inflammatory changes to the colon. In addition, infection with *C. jejuni* was also promoted by co-infection with entero-invasive *Escherichia coli* but unaffected over time. In contrast to *S. typhimurium* and entero-invasive *E. coli*, co-infection by *Listeria monocytogenes* showed little effect on *C. jejuni* infection and even hindered its progress. In addition, the intestinal microecology was also affected by co-infection of *C. jejuni* with other pathogens, with an increased relative abundance of unclassified Enterobacteriaceae, decreased levels of butyric acid and changes in the abundance of several genera of gut microbe, which suggests that some food-borne pathogenic bacteria might affect the progression of *C. jejuni* infection in mice by influencing the composition of the gut microbiota and the resulting changes in SCFA levels. Collectively, our findings suggest that co-infection of *Campylobacter* with other pathogenic bacteria can impact on the progression of infection by *C. jejuni* in mice, which may also have implication for the etiology of *Campylobacter* on human health.

## Introduction

*Campylobacter* is a food-borne pathogen and the leading cause of human gastroenteritis around the world. The incidence of campylobacteriosis has been estimated to be 4.4 per 1000 people, with 1.3 million cases in the United States (Scallan et al., 2011), 5.8 per 1000 person–years in the Netherlands (Havelaar et al., 2012), and 1.2 per 1000 person–years in China (Huang et al., 2018). *Campylobacter* infection usually results in symptoms such as bloody diarrhea, abdominal pain and fever, and the course of the disease is self-limited in most cases. However, an increased risk of irritable bowel syndrome and inflammatory bowel disease was observed amongst those infected with *Campylobacter* (Marshall et al., 2006). Moreover, some peripheral neuropathies such as Guillain–Barré syndrome and Miller Fisher syndrome are also long-term consequences of *Campylobacter* infection (Poropatich et al., 2010; Sejvar et al., 2011).

*Campylobacter* is most frequently found in poultry, but it can also be found in other food items such as pork, beef, and raw milk. The prevalence of *Campylobacter* contamination in retail poultry and by-products exceeds 50% around the world, varying from 0 to 100% (Sahin et al., 2015). In many developed countries, the rate of *Campylobacter* contamination usually exceeds 60%, but it can be much lower in developing countries such as China and Brazil (Silva et al., 2017; Zhu et al., 2017). Notably, when food items are contaminated with *Campylobacter*, they may also be contaminated with other food-borne pathogens. Many reports have found food samples that harbor more than one species of pathogen. In Ireland, 10 of 25 raw chicken samples were contaminated with multiple pathogens (Gorman et al., 2002). Another study also described the presence of more than two pathogens in ready-to-eat meat products (Gibbons et al., 2006). In addition, *Campylobacter* strains from different sources can also be detected in the same poultry house (Hiett et al., 2002). Due to the conventional intestinal microbiota, *Campylobacter* cannot effectively colonize mice sufficiently to exert obvious clinical symptoms (Dorrell and Wren, 2007). Acute ileitis induced by *Toxoplasma gondii* can abrogate the colonization resistance of mice, after which high loads of *Campylobacter* can be achieved (Haag et al., 2012a). However, it remains unclear whether other co-infection of pathogenic bacteria with *Campylobacter* can exacerbate the campylobacteriosis. To date, few studies have focused directly on campylobacteriosis caused by *Campylobacter* and other pathogenic bacteria.

In this study, mice were co-infected with *Campylobacter jejuni* and other pathogenic bacteria [*Salmonella typhimurium*, entero-invasive *Escherichia coli* (EIEC) and *Listeria monocytogenes*] to determine whether this multiple infection would lead to more severe campylobacteriosis in mice. Moreover, the changes in the microbiota and the level of short-chain fatty acids (SCFAs) in feces were also monitored to determine if multiple infections also affect the intestinal microenvironment which may explain the potential mechanism by which co-infection with food-borne pathogenic bacteria influences the progression of *C. jejuni* infection in mice.

## Materials and Methods

### Bacterial Strains and Culture Conditions

*Campylobacter jejuni* NCTC 11168 (ATCC 700819), *S. typhimurium* SL1344, EIEC (ATCC 43893), and *L. monocytogenes* (ATCC 19114) were acquired from the Culture Collection of Food Microorganisms of Jiangnan University (Wuxi, China). Columbia blood agar base plates (Oxoid, United Kingdom) supplemented with 5% sterile sheep blood and *C. jejuni* selective supplement (Oxoid) were used to culture *C. jejuni* strains under microaerophilic conditions (5% O_2_, 10% CO_2_, 85% N_2_) for 48 h at 37°C. *S. typhimurium*, EIEC and *L. monocytogenes* were cultured with brain-heart infusion broth (Haibo, China) for 24 h at 37°C.

### Animals and Experimental Design

Three-week-old female C57BL/6 mice were obtained from Shanghai Laboratory Animal Center (Shanghai, China) and used in all animal experiments. Six mice were housed in each cage, with a 12 h light–dark cycle in a controlled environment (temperature, 22 ± 2°C; humidity, 50 ± 5%). All experimental procedures (#JIPD2017029) were approved by the Animal Care and Use Committee at Jiangsu Institute of Parasitic Diseases. All experiments conformed to the China Ministry of Science and Technology Guide for the Care and Use of Laboratory Animals.

Mice were infected with pathogenic bacteria or parasites by gavage at a volume of 0.2 mL. *C. jejuni* concentration was adjusted to 2 × 10^9^ colony-forming units (CFU)/mL in sterile phosphate-buffered saline solution (PBS), and *S. typhimurium*, EIEC, *L. monocytogenes* were used at doses of 1 × 10^5^ CFU/mL in sterile PBS. Cysts of the *T. gondii* ME49 strain (from Jiangsu Institute of Parasitic Diseases Remington, Wuxi, China) were obtained from the homogenized brains of mice infected with 10 cysts for 2 months, and the mice were infected perorally with 100 *T. gondii* cysts. Sterile PBS was used as a naive control. The mice were divided into 11 groups; the treatment of each group is shown in **Table 1**. Each mouse’s body weight was recorded every 3 days, and stool samples were collected during the experimental procedure. The mice were anesthetised before sacrifice with an injection of 100 mg/kg body weight of ketamine, and plasma and colonic tissues were collected for further analysis.

**Table 1:** Animal model experimental design.

Groups	Treatment of different days				
	**Day 1**	**Day 2–4**	**Day 5–6**	**Day 7–10**	**Day 11**
Naive	PBS	Normal breed	PBS	Normal breed	Sacrificed
Cj	PBS		*C. jejuni*		
T.g + Cj	*T. gondii*		*C. jejuni*		
S.t + Cj	*S. typhimurium*		*C. jejuni*		
EIEC + Cj	EIEC		*C. jejuni*		
L.m + Cj	*L. monocytogenes*		*C. jejuni*		
T.g	*T. gondii*		PBS		
S.t	*S. typhimurium*		PBS		
EIEC	EIEC		PBS		
L.m	*L. monocytogenes*		PBS		

### Detection of *C. jejuni* in Feces

Stool samples were collected from all mice and transported to the laboratory on an ice pack. The feces were resuspended in sterile PBS and serially diluted. Diluted samples were spread on Columbia blood agar with *C. jejuni* selective supplement and incubated under microaerobic conditions at 37°C for 48 h. After incubation, *C. jejuni* numbers were determined by CFU.

### Bloody Stool Assay

A bloody stool detection kit (Jiancheng, China) was used to assess the presence of blood in fecal samples, which acted as a clinical sign of *C. jejuni*-induced infection. The detection kit was used immediately after the fecal samples were collected, and the level of bloody stool was divided into four grades according to a previous study (Siegmund et al., 2001).

### Determination of Colon Histopathology

The colonic samples were fixed in 4% neutral buffered paraformaldehyde and embedded in paraffin. Sections (5 μm) were stained with haematoxylin and eosin for light microscopic examination (magnification, ×100). The degree of inflammation and damage was evaluated using a histopathological score system (Paclik et al., 2008), modified as follows:

Score 0: Histological findings identical to those of normal mice.
Score 1: Loss of goblet cells begins.
Score 2: Single isolated cell infiltrates within mucosa; loss of goblet cells begins.
Score 3: Mild scattered to diffuse cell infiltrates within mucosa and submucosa; loss of goblet cells begins.
Score 4: Mild scattered to diffuse cell infiltrates within mucosa and submucosa; loss of goblet cells.
Score 5: Severe inflammation; loss of goblet cells; loss of crypts.
Score 6: Severe inflammation; loss of goblet cells; extensive ulceration.

This analysis was performed blind by a pathologist.

### Cytokine Assay

Blood samples were collected and centrifuged (1200 × *g*, 15 min) to obtain serum. Before cytokine assays, serum samples were treated using a Milliplex MAP Kit (Merck, Germany) according to the manufacturer’s instructions. A Luminex MAGPIX system (Luminex, United States) was used to detect the levels of cytokines in the treated serum samples.

### DNA Extraction, PCR, and 16S rDNA Sequencing

The fecal samples were stored at −80°C before detection, and microbial genome DNA was extracted using a FastDNA Spin Kit for Soil (MP Biomedical, United States) following the manufacturer’s instructions. The V3–V4 region of the 16S rRNA gene was amplified by PCR. The products were separated in 1.5% (w/v) agarose gel, purified with a QIAquick Gel Extraction Kit (Qiagen, Germany) and quantified with a Quant-iT PicoGreen dsDNA Assay Kit (Life Technologies, United States). A TruSeq DNA LT Sample Preparation Kit (Illumina, United States) was used to establish libraries that were sequenced for 500 + 7 cycles on Illumina MiSeq using a MiSeq Reagent Kit. QIIME pipeline was used to analyze the sequence data of 16S rRNA as described previously (Wang et al., 2017).

### Short-Chain Fatty Acid Assay

Fecal samples stored at −80°C were steeped in saturated NaCl solution for 30 min using sterile tubes and homogenized. Sulfuric acid (10%; 20 μL) was added to acidify the solution. Diethyl ether (800 μL) was added with an injection syringe to extract SCFAs. The tubes were centrifuged at 14,000 rpm for 15 min, and the supernatants collected. Anhydrous sodium sulfate was used to eliminate the remaining water, and the treated supernatants were analyzed using gas chromatography-mass spectrometry (GC-MS). The parameters for GC-MS in our experiment were established with reference to Sun et al. (2015).

### Statistical Analysis

Statistical analyses were performed using GraphPad Prism 5, and the data are expressed as mean ± SD. SPSS 20.0 (SPSS Inc., United States) was used for significance analysis. Comparisons between groups were made with a two-tailed Student’s *t*-test, and a two-sided *p*-value of less than 0.05 was considered to indicate statistical significance.

## Results

### Co-infection of *S. typhimurium* Elevated the *C. jejuni* Load in Mice

**FIGURE 1:**
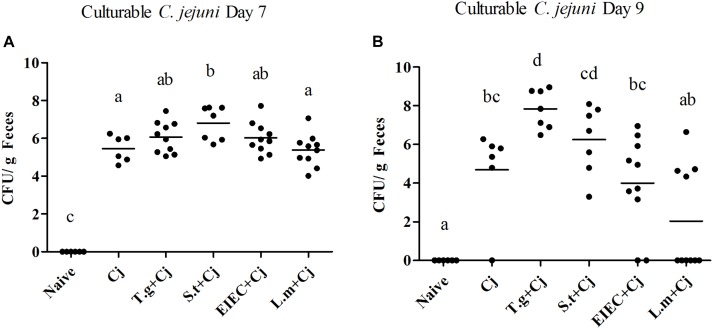
Culturable *C. jejuni* in feces when mice were infected by *C. jejuni* alone or together with other pathogen. **(A)** Culturable *C. jejuni* on day 7. **(B)** Culturable *C. jejuni* on day 9. Means with no common letters differ significantly (*P* < 0.05).

As shown in **Figure 1**, feces from all infected groups (*C. jejuni*, *T. gondii*+ *C. jejuni*, *S. typhimurium* + *C. jejuni*, EIEC + *C. Jejuni*, and *L. monocytogenes* + *C. jejuni*) were positive for *C. jejuni*, whilst *C. jejuni* was not detected in the mice from the control group (naive, *T. gondii*, *S. typhimurium*, EIEC, and *L. monocytogenes*). *T. gondii* infection led to significantly higher *C. jejuni* loads on day 9 than *C. jejuni* infection alone (*P* < 0.05). In addition, in the *T. gondii* coinfection group, more culturable *C. jejuni* was detected at day 9 than at day 7, and the number of viable bacteria reached 10^9^ CFU per gram of feces. *S. typhimurium* infection also aggravated *C. jejuni* colonization on day 7 in comparison with *C. jejuni* alone (*P* < 0.05), which was more effective than *T. gondii*, with the number of *C. jejuni* reaching 10^8^ CFU per gram of feces. On day 9, although *C. jejuni* loads in a few mice co-infected with *S. typhimurium* decreased, the loads remained mostly stable in other mice, where *C. jejuni* loads could reach 10^8^ CFU per gram of feces. In contrast, co-infection with EIEC or *L. monocytogenes* did not significantly enhance the *C. jejuni* loads on days 7 or 9 (*P* > 0.05). Compared with the overall increase of *C. jejuni* loads by *T. gondii*, the effects on *C. jejuni* colonization by three food-borne pathogens were distinct and pathogen specific. More individuals co-infected with *S. typhimurium* maintained the high *C. jejuni* loads. A few individuals co-infected with EIEC still had a higher *C. jejuni* load. However, the *C. jejuni* in most individuals co-infected with *L. monocytogenes* cleared, indicating the potential negative impact of *L. monocytogenes* on *C. jejuni* colonization.

### *S. typhimurium* Decreased the Body Weight of Mice When Co-infected With *C. jejuni*

**FIGURE 2:**
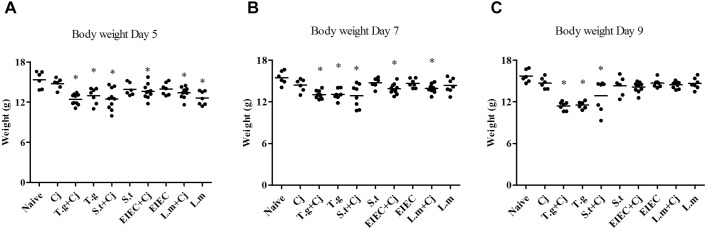
Body weight of mice over the course of infection. **(A)** Day 5. **(B)** Day 7. **(C)** Day 9. Asterisk (^∗^) indicates means that differ significantly from the naive group (*P* < 0.05).

Severe pathogenic infection can cause intestinal inflammation in mice, followed by a decrease in body weight. **Figure 2** shows that no significant decrease occurred in the body weights of the mice in the *C. jejuni* infection group. Consistent with previous reports (Grainger et al., 2013), the mice in the *T. gondii* only group and those in the *T. gondii*+ *C. jejuni* group showed significant decreases in body weight compared with the naive control group (*P* < 0.05), indicating that *T. gondii* infection seriously damaged their health status. Infection with three food-borne pathogens alone caused little change in the mice’s body weights. However, after co-infection with *C. jejuni*, the mice with *S. typhimurium* showed significant decreases in body weight compared with healthy mice; mice in the EIEC+ *C. jejuni* group only showed significant weight loss (*P* < 0.05) on days 5 and 7; mice in the *L. monocytogenes* and *L. monocytogenes* + *C. jejuni* groups only showed slight weight loss on day 5, but this effect completely reversed by day 9. It is noteworthy that, unlike the overall decrease in body weight in the *T. gondii*+ *C. jejuni* group, the decrease in mice body weight in the *S. typhimurium* + *C. jejuni* group showed obvious individual differences. Similar differences can also be seen in body weight gain (**Supplementary Figure S1** and **Supplementary Table S1**). On day 5, except for the mice in the *C. jejuni* group, all of the mice in the pathogen infection groups showed significantly reduced weight gain. The rate of weight gain recovered in all of the groups infected or co-infected with EIEC or *L. monocytogenes*. Although there were no significant differences between the weight gain of the *S. typhimurium* infected (and co-infected) groups and that of the naive group on day 9, obvious individual differences in the weight gain of the mice within this group were observed. The weight gain of each mouse corresponded to the *C. jejuni* load. In addition, several mice died in the *S. typhimurium* + *C. jejuni*, and in the *T. gondii*+ *C. jejuni* groups.

### Co-infection With *S. typhimurium* and *C. jejuni* Promoted Bloody Stools in Mice

**FIGURE 3:**
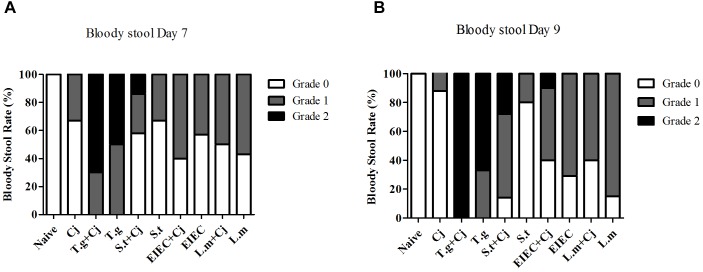
Occurrence of blood in fecal samples of mice over the course of infection. **(A)** Bloody stool rate on day 7. **(B)** Bloody stool rate on day 9.

Mice infected with *C. jejuni* had mild blood-positive feces by day 7, and the symptoms were partly relieved by day 9 (**Figure 3**). In *T. gondii*+ *C. jejuni* and *T. gondii* only groups, up to 50% of infant mice presented severe blood-positive (Grade 2) feces samples by days 7 and 9. In general, bloody stools were significantly more frequent in all co-infected groups than in groups infected with a single pathogen (either *C. jejuni* or another pathogenic bacteria), except that the bloody stools in the *L. monocytogenes* + *C. jejuni* group were less frequent than those in the *L. monocytogenes* group by day 9. Severe blood-positive (Grade 2) fecal samples were also observed in the *S. typhimurium* co-infection group (days 7 and 9) and in the EIEC coinfection group (day 9). It is noteworthy that although the frequency of bloody stools in the *S. typhimurium* only group was similar to or even lower than that in the EIEC and *L. monocytogenes* groups, the bloody stools in the *S. typhimurium* + *C. jejuni* group were more serious than those in all of the other co-infected groups, whilst the frequency of bloody stool caused by *S. typhimurium* infection was reduced to a relatively low level between 7 and 9 day.

### *C. jejuni* Relieved the Damage to Colonic Tissue Caused by Co-infected Food-Borne Pathogenic Bacteria

**FIGURE 4:**
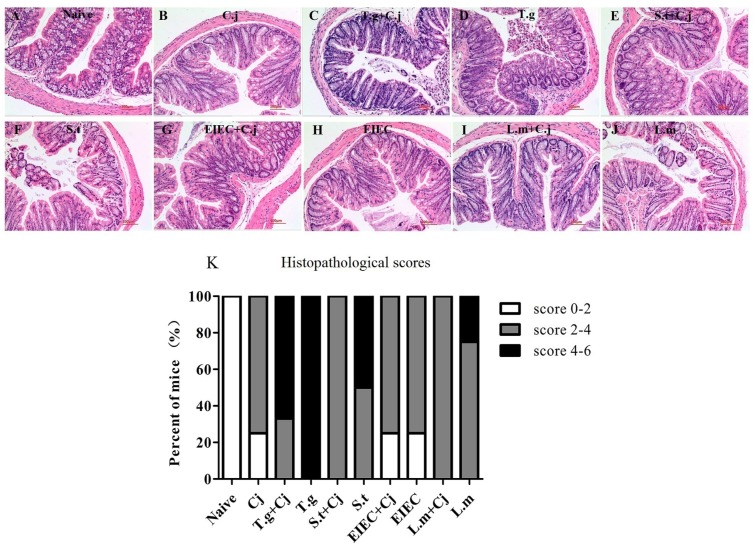
Histopathological changes in haematoxylin and eosin–stained colonic paraffin sections. **(A)** Naive group. **(B)** *C. jejuni* group. **(C)** *T. gondii* + *C. jejuni* group. **(D)** *T. gondii* group. **(E)** *S. typhimurium* + *C. jejuni* group. **(F)** *S. typhimurium* group. **(G)** EIEC + *C. jejuni* group. **(H)** EIEC group. **(I)** *L. monocytogenes* + *C. jejuni* group. **(J)** *L. monocytogenes* group. **(K)** Histopathological scores of colonic paraffin sections.

After the mice were killed (day 11), colon samples were collected from each group for histological analysis. As shown in **Figure 4A**, the mice from the naive group displayed normal villi, crypts, and muscular layer with a large number of goblet cells. Mice infected with *C. jejuni* alone had fewer goblet cells, whilst the structure of villi, crypts, and muscular layer was normal (**Figure 4B**). Infection with *T. gondii* alone resulted in severe colonic pathological changes, including a loss of goblet cells, damage to the crypt and villi architecture and inflammatory cell infiltration (**Figure 4D**), indicating that *T. gondii* infection induced serious inflammation and tissue injury. However, the histopathological scores in **Figure 4K** indicate that co-infected *C. jejuni* partly relieved the damage to the colonic tissue. *S. typhimurium* infection alone caused the highest histopathological scores in the groups of food-borne pathogenic bacteria infection alone, whilst EIEC caused the lowest scores, similar to that of *C. jejuni* (**Figures 4F,H,J**). It is noteworthy that, like the *T. gondii*+ *C. jejuni* group (**Figure 4C**), co-infected *C. jejuni* partly relieved the damage to the colonic tissue caused by *S. typhimurium* and *L. monocytogenes*, whilst it had no effects on the damage caused by EIEC (**Figures 4E,G,I**).

### Effects of Co-infected Food-Borne Pathogenic Bacteria on Inflammation

**FIGURE 5:**
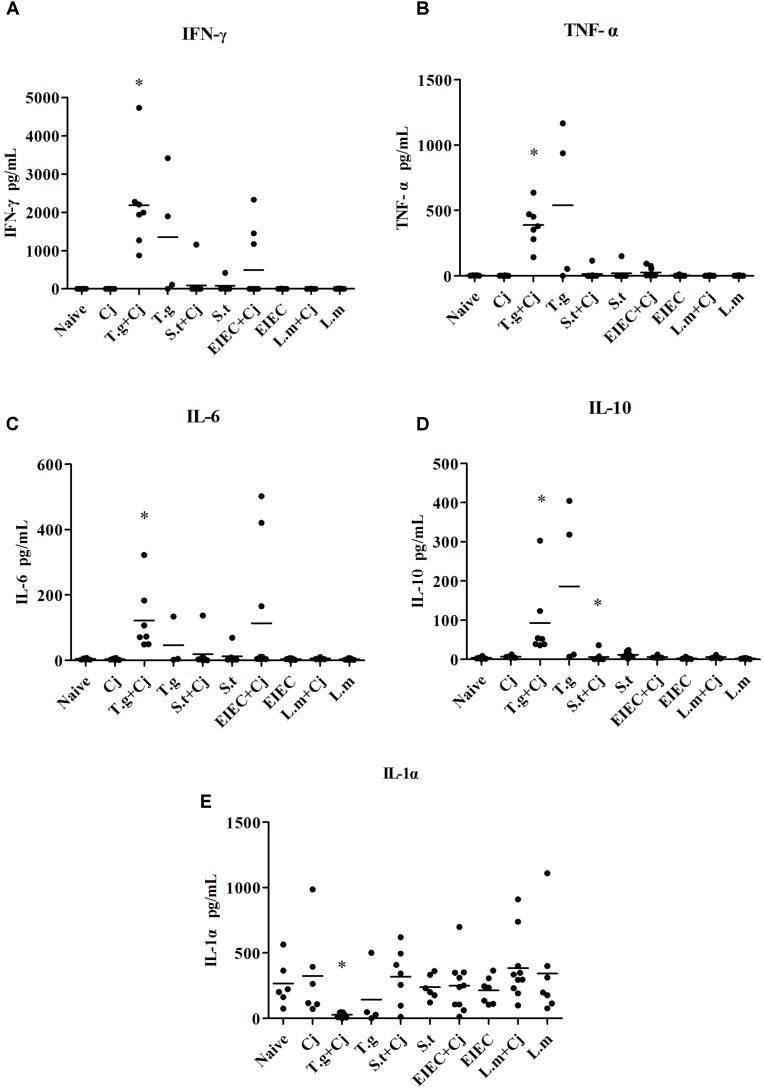
Cytokine production in infected mice. **(A)** IFN-γ. **(B)** TNF-α. **(C)** IL-6. **(D)** IL-10. **(E)** IL-1α. Asterisk (^∗^) indicates means that differ significantly from the naive group (*P* < 0.05).

The concentration of inflammatory factors in blood serum was also investigated. As shown in **Figure 5**, infection with *C. jejuni* did not induce any of the inflammatory cytokines. However, the concentrations of interferon (IFN) γ, tumour necrosis factor (TNF) α, interleukin (IL) 6, and IL-10 were significantly elevated due to infection with *T. gondii* alone, whilst the levels of IL-1α were down-regulated compared with other groups. In the *T. gondii*+ *C. jejuni* group, the concentrations of IFN-γ and IL-6 were higher than those in the *T. gondii* only group, whilst the concentrations of TNF-α, IL-10, and IL-1α were lower than those in the *T. gondii* group. As in the *T. gondii*+ *C. jejuni* group, the levels of IFN-γ, TNF-α, and IL-6 were also increased in some mice co-infected with *C. jejuni* in the EIEC + *C. jejuni* group, although no changes in any of the mice in the EIEC group. Moreover, IFN-γ, TNF-α, IL-10, and IL-6 in some mice in the *S. typhimurium* and *S. typhimurium* + *C. jejuni* groups also showed slight increases, although these were not significant. No significant variation could be detected in other cytokines, such as IL-1β, IL-2, IL-4, IL-12, and IL-17 (data not shown).

### Co-infection of Food-Borne Pathogenic Bacteria Alter the Composition of SCFAs in Feces

**FIGURE 6:**
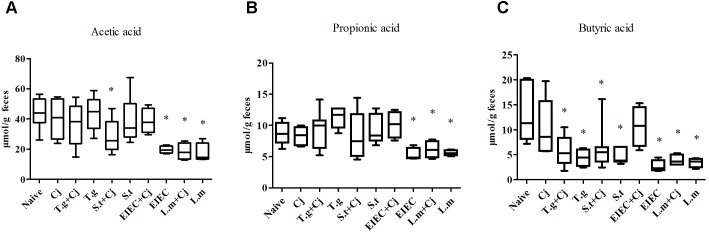
Level of SCFAs in feces of infected mice. **(A)** Acetic acid level. **(B)** Propionic acid level. **(C)** Butyric acid level. Asterisk (^∗^) indicates means that differ significantly from the naive group (*P* < 0.05).

The contents of acetic acid, propionic acid and butyric acid in feces were analyzed by GC-MS to evaluate the metabolism of the intestinal microbiota. **Figure 6** shows that *C. jejuni* infection alone did not cause significant changes in the composition of SCFAs in mouse feces compared to that in the naive group. In contrast, infection by EIEC or *L. monocytogenes* alone resulted in distinct decreases in the levels of acetic acid, propionic acid and butyric acid in mouse feces (*P* < 0.05). Interestingly, co-infection with *C. jejuni* led to recovery of the SCFA level in the EIEC group but showed no effects on the SCFA level in the *L. monocytogenes* group, indicating the specific alteration of microbial metabolism. Infection with *T. gondii* or *S. typhimurium* alone only decreased the level of butyric acid (*P* < 0.05). Co-infection with *C. jejuni* showed no significant effects on the SCFAs level influenced by *T. gondii* or *S. typhimurium* except for a further decrease in the acetic acid level in the *S. typhimurium* + *C. jejuni* group (*P* < 0.05).

### Co-infection of Food-Borne Pathogenic Bacteria Alter the Diversity and Relative Abundance of the Gut Microbiota

**FIGURE 7:**
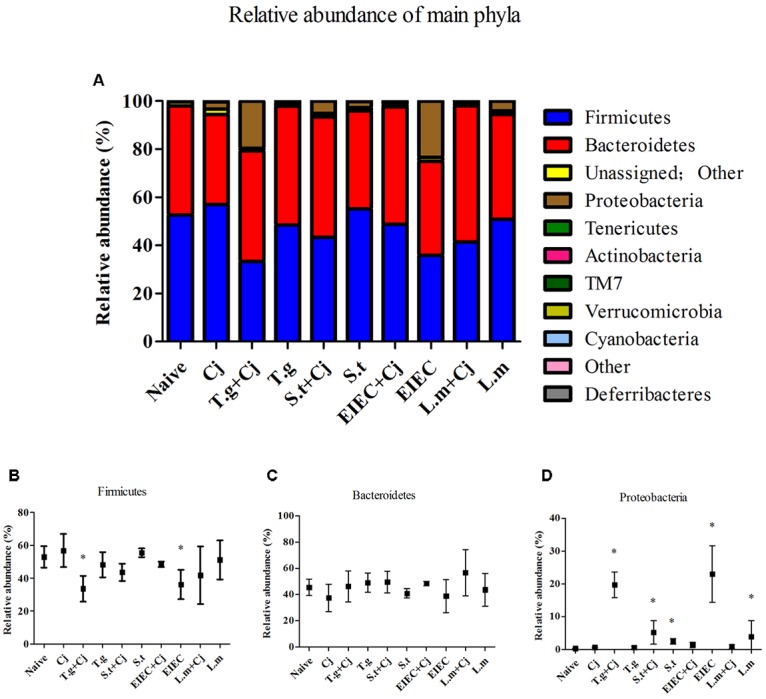
Microbial composition at phylum level in different groups. **(A)** Relative abundance of main phyla in different groups. **(B)** Firmicutes. **(C)** Bacteroidetes. **(D)** Proteobacteria. Asterisk (^∗^) indicates means that differ significantly from the naive group (*P* < 0.05).

The estimated richness (Chao-1) and Shannon index were used to evaluate the community diversity of each sample. **Supplementary Figure S2** shows that infection by *C. jejuni* alone seemed to cause no obvious differences in the diversity of gut microbiota. Infection by *T. gondii*, *S. typhimurium*, EIEC, or *L. monocytogenes* alone caused significant decreases in gut microbiota diversity. In addition, there was a tendency toward further reduction in the diversity of the gut microbiota in mice co-infected with *C. jejuni*, although individual differences existed between different mice. At phylum level (**Figure 7**), Firmicutes, Bacteroidetes, and Proteobacteria were dominant amongst the experimental groups. Tenericutes, Actinobacteria, TM7, Verrucomicrobia, and Cyanobacteria were also found in the feces of mice with a relative abundance of less than 1%. Mice co-infected with *T. gondii* and *C. jejuni* and those infected with EIEC alone showed a decrease in the relative abundance of Firmicutes compared with the naive group (*P* < 0.05). Each experimental group had between 20 and 40% Bacteroidetes in feces; no statistical differences were seen (*P* > 0.05). Notably, infection by different pathogens induced evident changes in Proteobacteria. The relative abundance of Proteobacteria in the naive control group was less than 1% but increased significantly in the *T. gondii* + *C. jejuni*, the *S. typhimurium* + *C. jejuni*, the *S. typhimurium*, EIEC, and the *L. monocytogenes* groups. The abundances of Proteobacteria in the *T. gondii* + *C. jejuni*, the *S. typhimurium* + *C. jejuni*, the *S. typhimurium* and the EIEC groups were positively correlated with the corresponding pathogen loads in the intestine. Although *L. monocytogenes* is not a member of the Proteobacteria, it significantly increased the abundance of Proteobacteria in the gut. Interestingly, although EIEC and *L. monocytogenes* increased the abundance of Proteobacteria, the presence of *C. jejuni* significantly decreased the abundance of this phylum, indicating an acceleration in the elimination of pathogenic bacteria from the host in the late period of co-infection.

**FIGURE 8:**
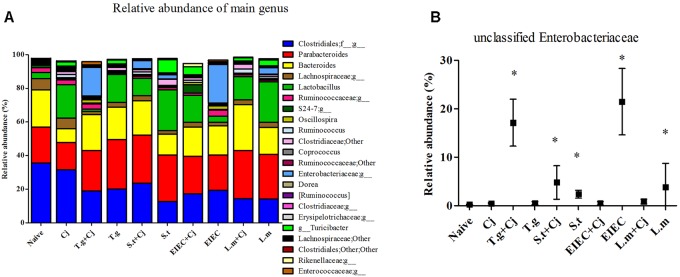
Microbial composition at genus level in different groups. **(A)** Relative abundance of main genus in different groups. **(B)** Unclassified Enterobacteriaceae. Asterisk (^∗^) indicates means that differ significantly from the naive group (*P* < 0.05).

The microbial composition was further analyzed at the genus level, to further explore the differences observed in the Proteobacteria phylum. **Figure 8A** shows all genera with a relative abundance of more than 1%. Unclassified genera within Enterobacteriaceae is the only genus in the genera listed in **Figure 8A** that belongs to Proteobacteria phylum. The relative abundance of unclassified Enterobacteriaceae in the experimental groups was then checked, and significant increases in unclassified Enterobacteriaceae were found in the *T. gondii* + *C. jejuni*, *S. typhimurium* + *C. jejuni*, the *S. typhimurium*, the EIEC, and the *L. monocytogenes* groups (*P* < 0.05), which exhibited the same tendency as Proteobacteria phylum (**Figure 8B**). This indicates that all four food-borne pathogenic bacteria can increase the abundance of unclassified Enterobacteriaceae. In addition to changes in the levels of unclassified Enterobacteriaceae, decreases in the abundance of unclassified Clostridiales and Lachnospiraceae corresponded to exposure of pathogenic bacteria, except no significant reduction in abundance was observed in the group infected with *C. jejuni* alone (**Supplementary Figure S3**). The abundance of these two genera also showed positive correlations with the diversity of the gut microbiota and the SCFA level. In addition, although low *C. jejuni* loads in the *C. jejuni* group caused no significant changes in the different indexes, the abundances of *Bacteroides* and *Lactobacillus* were significantly changed by infection with *C. jejuni* alone. The abundance of *Turicibacter* was also relatively high in the two co-infection groups with high *C. jejuni* loads. Moreover, although large individual differences were observed, the abundances of *Dorea* and unclassified S24-7 showed significant increases only in some of the mice in the EIEC + *C. jejuni* group, and were highly correlated with the recovery of SCFA level in this group.

## Discussion

Although many efforts have been made to prevent *C. jejuni* infections, it remains the most common food-borne pathogen from a global perspective (Haagsma et al., 2013). Humans can become infected with *C. jejuni* via contaminated water and food and by direct contact (Domingues et al., 2012). However, these pathways are compatible for other food-borne pathogenic bacteria as well, and the shared pathways lead to cross contamination. Contamination by multiple pathogenic bacteria, including different strains of *Campylobacter* from different sources (Konkel et al., 2007; Atterby et al., 2018), may cause symptoms that are more complex and serious than those caused by infection by only one pathogen. This study was performed to investigate whether co-infection with other common pathogenic bacteria affects the symptoms caused by *C. jejuni*. The results of our study show that co-infection with *S. typhimurium* significantly increased the *C. jejuni* load and resulted in more severe symptoms of *C. jejuni* infection. EIEC promoted infection by *C. jejuni* to some extent, but the promotion effects subsided with time. Co-infection with *L. monocytogenes* had no effects on *C. jejuni* load and even showed some reduction. Moreover, during co-infection with pathogenic bacteria, variations in the abundance of gut microbes such as unclassified Enterobacteriaceae, Clostridiales, and Lachnospiraceae, and corresponding changes in the level of SCFAs in the gut were observed (**Supplementary Tables S2–S6**).

*Campylobacter jejuni* has the disadvantage of sporadic colonization and barely triggers disease-defining clinical manifestations in mice due to their well established robust intestinal microbiota. In this study, 3-week-old female C57BL/6 mice with reported susceptibility to *C. jejuni* were used (Field et al., 1981). However, *C. jejuni* colonization levels remained low and even decreased beyond the infection day, which is consistent with the colonization resistance reported previously (Heimesaat et al., 2013). In addition, no significant pathological symptoms were found with *C. jejuni* alone, which may be related to the low colonization by *C. jejuni*. We therefore used *T. gondii* infection model (Liesenfeld, 2002; Haag et al., 2012b), that make the host more susceptible to *C. jejuni* infection as a positive control to assess the impact of food-borne pathogens on *C. jejuni* infections.

Because infection with high doses of *S. typhimurium*, EIEC, and *L*. *monocytogenes* can result in loss of weight, enteritis, and death in a mouse model (Miller and Burns, 1970; Medici et al., 2005; Martins et al., 2013; Franca et al., 2015), these food-borne pathogens are expected to promote the infection of *C. jejuni*. However, our study showed that only co-infection with *S. typhimurium* can promote *C. jejuni* colonization, and induce weight loss and bloody stools, even with a low infectious dose (3 × 10^4^ CFU/mouse) of *S. typhimurium*. Bloody stools and weight loss can be caused by *C. jejuni* alone to some extent (Haag et al., 2012a; Heimesaat et al., 2014). In the presence of *S. typhimurium*, *C. jejuni* was found to promote weight loss, bloody stools and even death of the mice. These results suggest that the presence of *S. typhimurium* can enhance *C. jejuni* numbers and exacerbate the corresponding symptoms. Although no significant difference in weight loss was found between the EIEC + *C. jejuni* group and the EIEC group, the bloody stools in the EIEC + *C. jejuni* group suggest that the presence of EIEC may promote *C. jejuni* infection to a limited extent. *L. monocytogenes* is a poor colonizer of the mouse intestine due to lack of recognition by mouse intestinal cell receptors (Lecuit et al., 1999) and germ free mice (Manohar et al., 2001) and mice pre-treated with streptomycin can increase the pathogen load (Takeuchi et al., 2006). However, even low-dose *L. monocytogenes* gavage still caused damage to the gut health of the mice. Unexpectedly, it seems that the symptoms in the *L. monocytogenes* + *C. jejuni* group were milder than those in the *L. monocytogenes* group. Taking the *C. jejuni* loads into consideration, it seems that the co-existence of these two pathogenic bacteria impedes each other’s infection process. Corresponding with the above results, histological analysis show that the mice infected with *C. jejuni* alone displayed mild pathological changes in the colon, likely due to sporadic colonization of *C. jejuni.* However, different to the results of weight loss and bloody stool, except for the EIEC group, it seems that the co-infected *C. jejuni* instead reduces the degree of intestinal tissue lesions. This finding may be related to the colonization area of the pathogens, and further investigation is needed.

Although the major site of *T. gondii* replication is different from that of the other four food-borne pathogenic bacteria (Dubey et al., 1997), *T. gondii* promoted infection with *C. jejuni* in mice. This indicates that the synergistic effect may not be confined to the site of infection but rather a systemic effect. *T. gondii* infection leads to Th1-type immunopathology in mice, which causes an elevated IFN-γ concentration (Heimesaat et al., 2007; Zhou and Wang, 2017). The effects of one pathogen on the host’s immune system may be the cause of the host’s susceptibility to or tolerance of other pathogens. IL-10-/- mice that show heavier pathogen loads and more severe clinical signs are usually used to establish models of *C. jejuni* infection (Bereswill et al., 2011; Sun et al., 2012). This indicates that changes in the host’s immune status may have an important impact in *C. jejuni* infection. It has been reported that TNF-α, IL-1, IL-4, and IL-10 are all elevated in mice after *C. jejuni* infection (Al-Banna et al., 2012). In this study, co-infection of *T. gondii* and *C. jejuni* further increased the levels of IFN-γ and IL-6 caused by *T. gondii*, much in the same way as the changes in cytokines induced by *C. jejuni* in gnotobiotic mice (Bereswill et al., 2011), which suggests that *T. gondii* infection promotes *C. jejuni* infection symptoms. In addition, from the level of cytokines, it seemed that EIEC does promote *C. jejuni* infection to a certain extent, whilst *L. monocytogenes* infection without any promotion on *C. jejuni* infection, which is also confirmed by the disease indicators described above. However, unexpectedly, although *S. typhimurium* can promote *C. jejuni* infection and its related symptoms in mice from pathogen loads and disease index, and *S. typhimurium* may activate local inflammatory processes in the colon with elevated levels of IFN-γ, TNF-α, and IL-6 (Castillo et al., 2011), *S. typhimurium* infection, whether alone or together with *C. jejuni*, did not result in statistical changes in cytokine levels in this study, despite slight increases in IFN-γ and IL-6 in a few mice. This indicates that infection promotion on *C. jejuni* by *S. typhimurium* differs somewhat from that by *T. gondii*.

Studies have shown that in addition to the immune system, the colonization of *C. jejuni* in the intestine is closely related to the gut microbiota (Bereswill et al., 2011). Even in the C57/6J mice that were proven to be less susceptible to *C. jejuni* colonization, a small number of individuals with high *C. jejuni* loads also showed significant differences in the composition of their caecal microbiota. *C. jejuni* colonization did not incite visible pathologic changes, but was associated with increased abundances of Coriobacteriaceae, Lachnospiraceae, and Ruminococcaceae (Lone et al., 2013). This suggests that the gut microbiota might play an important role in determining the extent of which *C. jejuni* can colonize the mice gut. An investigation in humans also indicated that low diversity of gut microbiota may result in *C. jejuni* infection (Kampmann et al., 2016). In addition, infection with *S. typhimurium*, EIEC and other pathogens can lead to a decline in the diversity of the gut microbiota and a decrease in the abundance of some intestinal microbes (Chassaing et al., 2014; Mon et al., 2015; Borton et al., 2017; Zhang et al., 2017; Du et al., 2018). In this study, because of the low load of *C. jejuni*, no significant change in the diversity of gut microbiota was found in the group infected with only *C. jejuni*. However, all other groups treated with pathogens showed significant decreases in the diversity of the gut microbiota. Co-infection with *C. jejuni* generally resulted in a tendency toward further reduction of the diversity of the gut microbiota, suggesting that infection with some food-borne pathogens may increase the colonization rate of *C. jejuni* by decreasing the diversity of gut microbiota.

Firmicutes and Bacteroidetes are the two dominant phyla in the murine intestinal microbiota, and the abundance of these phyla is related to host health (Hansen et al., 2010). In this study, the two groups whose Firmicutes/Bacteroidetes ratio decreased also showed an increase in the relative abundance of Proteobacteria, a phylum including a wide variety of pathogens, such as *Escherichia*, *Salmonella*, *Vibrio*, *Helicobacter*, *Yersinia*, *Legionella*, and many other notable genera (Madigan et al., 2018). In addition, although no significant decrease was seen in the level of Firmicutes, the *S. typhimurium*, *S. typhimurium*+ *C. Jejuni*, and *L. monocytogenes* groups also showed significant increases in Proteobacteria. This might reflect the effective colonization of this three pathogen in the mice. Unexpectedly, the increase in Proteobacteria abundance caused by infection with EIEC or *L. monocytogenes* disappeared due to the presence of *C. jejuni*. This was further confirmed by the variation in abundance of unclassified Enterobacteriaceae at the genus level. Infection with some pathogens has been reported to increase the level of Enterobacteriaceae in the gut (Corridoni et al., 2012; David et al., 2014). The lack of change in unclassified Enterobacteriaceae abundance in the *T. gondii* group but significant increase in the *T. gondii*+ *C. jejuni* group in this study suggests that the increase in unclassified Enterobacteriaceae is a manifestation of *C. jejuni* infection (Sakaridis et al., 2018). The increased abundance of unclassified Enterobacteriaceae may also predicts the possibility of an elevated rate of *Salmonella* colonization in association with *C. jejuni* infection. However, for EIEC and *L. monocytogenes*, there appears to be an antagonistic relationship that makes *C. jejuni* and these two pathogens unable to co-exist at high levels in the host’s gut. This may be due to the different effects of these bacteria on the immune system, or differential effects on the composition of the gut microbiota.

Changes in the abundances of unclassified Clostridiales and Lachnospiraceae also showed similar patterns with respect to gut microbiota diversity. As the Clostridiales includes a portion of the intestinal bacteria producing butyric acid (Liu et al., 2010; Myszka et al., 2012; Cai et al., 2013), these decreases can also explain the general decline of intestinal butyric acid levels in infected mice. It has been reported that butyric acid has potent anti-inflammatory effects and can efficiently maintain the integrity of the intestinal mucosa (Mishiro et al., 2013). Therefore, infection with these pathogenic bacteria may cause intestinal damage by affecting the abundance of SCFA-producing bacteria in the intestinal tract. The decrease in unclassified Lachnospiraceae observed in this study was also consistent with previous studies (Kampmann et al., 2016), but no correlations of *Coprococcus* and *Dorea* abundances were found in our study. Furthermore, similar to *Dorea*, unclassified S24-7 also showed a significant increase in only some mice in the EIEC + *C. jejuni* group, consistent with the recovery of SCFAs in this group. Both of these genera have been reported to produce acetic, propionic and butyric acids (Taras et al., 2002; Ormerod et al., 2016; Meisel et al., 2017; Bishehsari et al., 2018). Therefore, the specific regulation of gut microbiota by EIEC and *C. jejuni*, resulting in the restoration of SCFA levels may also be a cause of infection remission. In addition, similar to the findings of a previous study (Borewicz et al., 2015), the levels of *Turicibacter* in this study also increased due to *S. typhimurium* or *T. gondii* infection, which correlates with the promotion of *C. jejuni* colonization by these two pathogens. It has been reported there was a significantly lower proportion of *Turicibacter* in *Tnf*-/- compared to WT mice both prior to and after colitis induction (Jones-Hall et al., 2015). According to the levels of TNF-α in this study, the changes in cytokine and *Turicibacter* abundance showed a certain degree of coincidence. Whether the increase in *Turicibacter* abundance and TNF-α levels contribute to colonization by *C. jejuni* still needs further study. Besides, the changes in the abundances of *Parabacteroides* and *Lactobacillus* showed similar tendency in this study. Taking the effects on the host by these genera into account (Sanchez et al., 2010; Clarke et al., 2011), these changes appear to be a self-protection by the host to alleviate further infections and injuries. However, this protection seems to be limited because the abundances of these genera decrease in seriously co-infected mice. Therefore, changes in the gut microbiota caused by pathogens would further affect aspects of the gut environment such as metabolites, nutrients, and immune factors, influencing the infection progression of subsequent pathogens.

## Conclusion

This study demonstrates that different food-borne pathogenic bacteria co-infected with *C. jejuni* exert different effects on the progress of *C. jejuni* infection in mice. Co-infection with *S. typhimurium* can significantly increase the *C. jejuni* burden in mice and lead to more severe campylobacteriosis. Moreover, co-infection with EIEC promotes infection by *C. jejuni* to some extent, but this promotion disappears over time. In contrast, co-infection with *L. monocytogenes* has little effect on *C. jejuni* infection and even hinders its progress. In addition, an increase in the relative abundance of Enterobacteriaceae and a decreased level of butyric acid were also observed during co-infection of *C. jejuni* with other pathogenic bacteria. Changes in the abundance of some intestinal microbes may be directly related to the progression of *C. jejuni* infection and they might thus be used as indicators of *C. jejuni* infection. Given the possibility of co-infection, it is clear from this study that some food-borne pathogenic bacteria might play an important role in the progression of *C. jejuni* infection.

## Author Contributions

GW, YZ, and WC conceived and designed the experiments. GW, YH, XJ, and XC performed the experiments. JZ and HZ analyzed the data. YZ, JZ, HZ, and WC contributed reagents, materials, and the analysis tools. GW and YH wrote the paper. All authors contributed to manuscript revision, read, and approved the submitted version.

## Conflict of Interest Statement

The authors declare that the research was conducted in the absence of any commercial or financial relationships that could be construed as a potential conflict of interest.
