# Supplementary material for: The Effect of Co-infection of Food-Borne Pathogenic Bacteria on the Progression of Campylobacter jejuni Infection in Mice
Source: Front Microbiol. 2018 Aug 22;9:1977. doi: 10.3389/fmicb.2018.01977 (PMC6113366; doi:10.3389/fmicb.2018.01977)
Supplement: Supplementary file 1 [file Data_Sheet_1.docx]

Supplementary Material

The effect of co-infection of food-borne pathogenic bacteria on the progression of *Campylobacter jejuni* infection in mice

**Gang Wang, Yufeng He, Xing Jin, Yonghua Zhou*, Xiaohua Chen, Jianxin Zhao, Hao Zhang, Wei Chen***

*** Correspondence: Yonghua Zhou & Wei Chen**

**zhouyonghua@jipd.com & chenwei66@jiangnan.edu.cn**

# Supplementary Figures and Tables

## Supplementary Figures
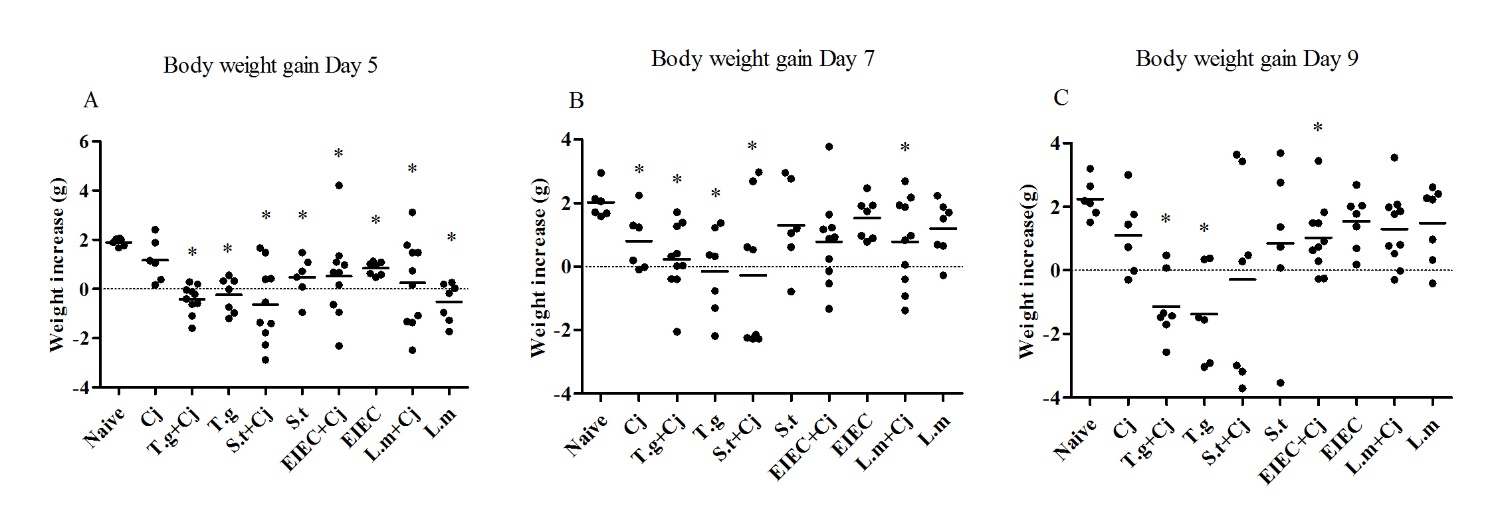


**Supplementary Figure 1.** Body weight gain of mice over the course of infection. (A) Day 5. (B) Day 7. (C) Day 9. Asterisk (*) indicates means that differ significantly from the naive group (*P* < 0.05).


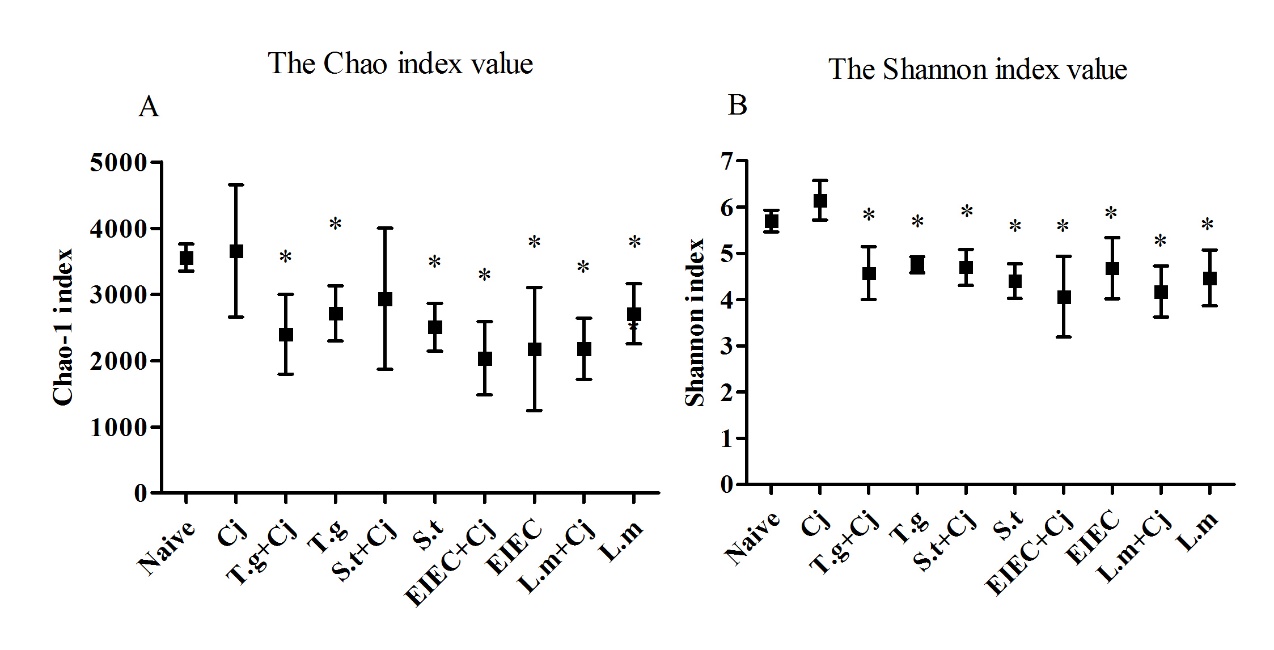


**Supplementary Figure 2.** Diversity of gut microbiota in each experimental groups. **(A)** Chao-1 index. **(B)** Shannon index. Asterisk (*) indicates means that differ significantly from the naive group (*P <* 0.05).


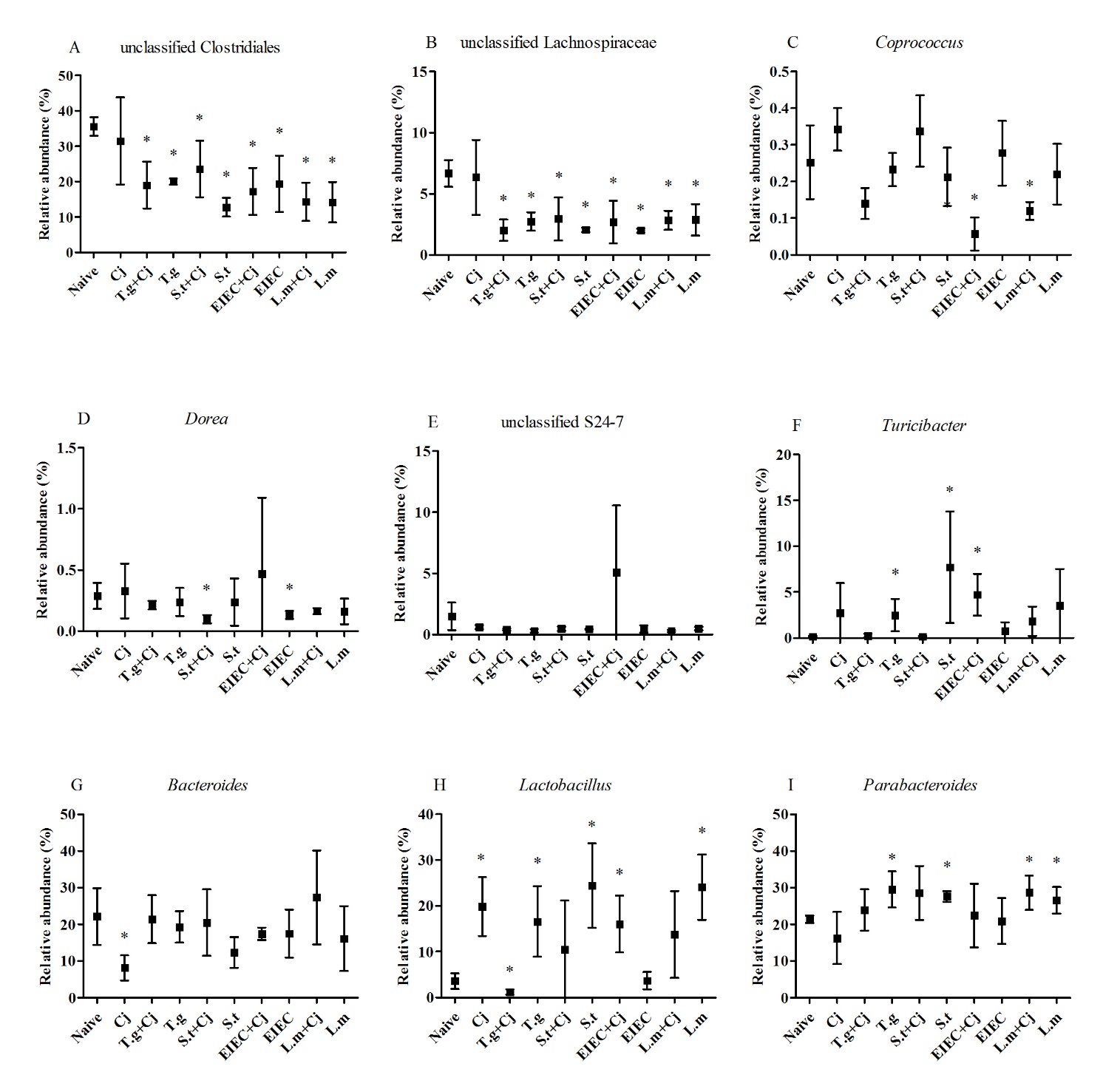


**Supplementary Figure 3.** Microbial composition at genus level in different groups. Asterisk (*) indicates means that differ significantly from the naive group (*P* < 0.05).

## Supplementary Tables

**Supplementary Table 1.** Body weight of each individual mouse over the course of infection.

| **Group / Number** | | **Body weight (g)** | | | | **Group / Number** | | **Body weight (g)** | | | |
| --- | --- | --- | --- | --- | --- | --- | --- | --- | --- | --- | --- |
|  |  | **Day1** | **Day5** | **Day7** | **Day9** |  |  | **Day1** | **Day5** | **Day7** | **Day9** |
| **Naive** | A | 13.16 | 15.17 | 14.87 | 14.98 | **Cj** | A | 13.07 | 14.95 | 12.97 | 14.83 |
|  | B | 14.49 | 16.55 | 16.62 | 16.68 |  | B | 13.10 | 14.25 | 14.33 | 13.83 |
|  | C | 14.73 | 16.49 | 16.41 | 16.85 |  | C | 14.72 | 14.88 | 14.91 | 14.42 |
|  | D | 14.08 | 16.08 | 15.66 | 15.59 |  | D | 12.85 | 15.26 | 15.09 | 15.85 |
|  | E | 12.00 | 13.89 | 14.06 | 14.65 |  | E | 12.41 | 13.46 | 13.70 | 13.85 |
|  | F | 12.14 | 13.81 | 15.08 | 15.34 |  | F | 15.32 | 15.70 | 15.30 | 15.31 |
| **T.g+Cj** | A | 11.97 | 11.86 | 12.29 | 10.63 | **S.t+Cj** | A | 13.91 | 12.51 | 11.67 | 10.92 |
|  | B | 14.68 | 13.08 | 12.63 | × |  | B | 12.22 | 9.95 | × | × |
|  | C | 13.01 | 11.91 | 13.04 | 11.58 |  | C | 11.00 | 12.47 | 13.96 | 14.64 |
|  | D | 11.13 | 11.09 | 12.39 | 11.21 |  | D | 13.08 | 11.72 | 10.80 | × |
|  | E | 11.34 | 11.62 | 13.05 | 11.81 |  | E | 13.70 | 10.81 | × | × |
|  | F | 11.62 | 11.82 | 13.00 | × |  | F | 11.14 | 12.80 | 13.82 | 14.57 |
|  | G | 13.52 | 12.89 | 13.54 | 11.82 |  | G | 14.71 | 14.16 | 12.57 | 11.53 |
|  | H | 13.60 | 13.20 | 14.00 | 12.13 |  | H | 13.96 | 14.38 | 14.57 | 14.44 |
|  | I | 14.00 | 13.41 | 13.60 | × |  | I | 13.00 | 11.22 | 10.72 | 9.29 |
|  | J | 13.19 | 12.97 | 12.80 | 10.62 |  | J | 14.23 | 14.62 | 14.76 | 14.51 |
| **T.g** | A | 12.71 | 12.70 | 13.03 | 11.15 | **S.t** | A | 11.68 | 13.16 | 14.44 | 14.44 |
|  | B | 14.77 | 14.03 | 14.00 | 11.86 |  | B | 14.49 | 15.20 | 15.54 | 15.23 |
|  | C | 13.68 | 14.00 | 14.04 | 12.20 |  | C | 14.91 | × | × | × |
|  | D | 14.98 | 13.78 | 12.79 | × |  | D | 12.31 | 13.39 | 15.26 | 16.00 |
|  | E | 10.45 | 11.00 | 11.82 | 10.79 |  | E | 12.92 | 13.00 | 13.53 | 13.00 |
|  | F | 14.34 | 13.37 | 13.03 | 11.30 |  | F | 13.31 | 13.79 | 14.51 | 14.67 |
|  | G | 11.45 | 11.77 | 12.67 | 11.83 |  | G | 15.89 | 14.94 | 15.10 | 12.35 |

| **EIEC+**  **Cj** | A | 12.22 | 12.88 | 13.44 | 13.71 | **L.m+Cj** | A | 13.80 | 12.72 | 13.40 | 14.60 |
| --- | --- | --- | --- | --- | --- | --- | --- | --- | --- | --- | --- |
|  | B | 11.51 | 15.72 | 15.28 | 14.95 |  | B | 12.48 | 13.95 | 14.65 | 14.55 |
|  | C | 14.21 | 13.57 | 13.68 | 14.5 |  | C | 13.03 | 13.77 | 14.00 | 13.81 |
|  | D | 14.14 | 13.19 | 14.00 | 14.78 |  | D | 11.70 | 13.48 | 13.63 | 13.69 |
|  | E | 12.80 | 13.47 | 13.97 | 14.28 |  | E | 14.20 | 12.83 | 14.26 | 14.73 |
|  | F | 12.56 | 12.72 | 13.48 | 13.48 |  | F | 13.22 | 13.37 | 14.05 | 15.08 |
|  | G | 12.83 | 14.18 | 13.07 | 12.56 |  | G | 11.16 | 14.27 | 13.84 | 14.71 |
|  | H | 12.70 | 13.67 | 14.34 | 14.53 |  | H | 14.68 | 13.35 | 13.75 | 14.38 |
|  | I | 13.65 | 14.74 | 14.52 | 14.39 |  | I | 12.99 | 14.46 | 14.86 | 14.76 |
|  | J | 14.07 | 11.76 | 12.74 | 13.82 |  | J | 14.09 | 11.60 | 12.71 | 14.07 |
| **EIEC** | A | 14.48 | 14.95 | 15.44 | 15.86 | **L.m** | A | 11.45 | 11.70 | 13.68 | 14.07 |
|  | B | 12.18 | 13.26 | 13.92 | 14.22 |  | B | 15.02 | 13.74 | 15.67 | 15.35 |
|  | C | 14.64 | 15.22 | 15.42 | 14.83 |  | C | 15.23 | 13.49 | 14.95 | 14.82 |
|  | D | 11.98 | 12.94 | 14.44 | 14.67 |  | D | 11.18 | 11.38 | 12.68 | 13.45 |
|  | E | 11.99 | 13.11 | 13.91 | 14.00 |  | E | 13.48 | 12.52 | 14.17 | 14.45 |
|  | F | 13.60 | 14.23 | 14.49 | 14.30 |  | F | 11.93 | 11.75 | 13.80 | 14.34 |
|  | G | 13.00 | 14.02 | 14.91 | 14.78 |  | G | 13.65 | 13.68 | 15.35 | 15.88 |

Mice died during infection are represented by X mark.

**Supplementary Table 2.** Infection symptoms associated with different pathogens treatment.

| **Group** | **Infection symptoms** | | | | | | | |
| --- | --- | --- | --- | --- | --- | --- | --- | --- |
|  | **Culturable Cj** | | **Body weight** | | | **Bloody stool** | | **Colonic damage** |
|  | Day7 | Day9 | Day5 | Day7 | Day9 | Day7 | Day9 |  |
| **Naive** | ND | ND | - | - | - | - | - | - |
| **Cj** | 2 | 1 | - | - | - | - | - | - |
| **T.g+Cj** | 3 | 4 | ↓ | ↓ | ↓ | ++ | ++ | ++ |
| **T.g** | ND | ND | ↓ | ↓ | ↓ | ++ | ++ | ++ |
| **S.t+Cj** | 3 | 3 | ↓ | ↓ | ↓ | ++ | ++ | + |
| **S.t** | ND | ND | - | - | - | + | + | ++ |
| **EIEC+Cj** | 3 | 1 | ↓ | ↓ | - | + | ++ | - |
| **EIEC** | ND | ND | - | - | - | + | + | - |
| **L.m+Cj** | 2 | 1 | ↓ | ↓ | - | + | + | + |
| **L.m** | ND | ND | ↓ | - | - | + | + | ++ |

Culturable *C. jejuni* in mice feces are shown in different scores: score 1 indicates less than 10^5^ CFU/g, score 2 between 10^5^ CFU/g and 10^6^ CFU/g, score 3 between 10^6^ CFU/g and 10^7^ CFU/g, score 4 indicates more than 10^7^ CFU/g, ND means not detected. For body weight assay, groups which show no significance compared with naive group are represented by “-”. Groups which significantly lower than naive group are represented by “↓”. For bloody stool assay, groups which only contain Grade 0 are represented by “-”, groups which contain Grade 1 but not Grade 2 are represented by “+”, groups which contain Grade 2 are represented by “++”. For colonic damage, groups which contain score 0-2 are represented by “-”, groups which contain score 2-4 are represented by “+”, groups which contain score 4-6 are represented by “++”.

**Supplementary Table 3.** Cytokines level associated with different pathogens treatment.

| **Group** | **Cytokines** | | | | |
| --- | --- | --- | --- | --- | --- |
|  | **IFN-γ** | **TNF-α** | **IL-6** | **IL-10** | **IL-1α** |
| **Naive** | - | - | - | - | - |
| **Cj** | - | - | - | - | - |
| **T.g+Cj** | ↑ | ↑ | ↑ | ↑ | ↓ |
| **T.g** | - | - | - | - | - |
| **S.t+Cj** | - | - | - | ↑ | - |
| **S.t** | - | - | - | - | - |
| **EIEC+Cj** | - | - | - | - | - |
| **EIEC** | - | - | - | - | - |
| **L.m+Cj** | - | - | - | - | - |
| **L.m** | - | - | - | - | - |

For cytokines assay, groups which show no significance compared with naive group are represented by “-”, groups which significantly lower than naive group are represented by “↓”, groups which significantly higher than naive group are represented by “↑”.

**Supplementary Table 4.** SCFAs level associated with different pathogens treatment.

| **Group** | **SCFAs** | | |
| --- | --- | --- | --- |
|  | **Acetic acid** | **Propionic acid** | **Butyric acid** |
| **Naive** | - | - | - |
| **Cj** | - | - | - |
| **T.g+Cj** | - | - | ↓ |
| **T.g** | - | - | ↓ |
| **S.t+Cj** | ↓ | - | ↓ |
| **S.t** | - | - | ↓ |
| **EIEC+Cj** | - | - | - |
| **EIEC** | ↓ | ↓ | ↓ |
| **L.m+Cj** | ↓ | ↓ | ↓ |
| **L.m** | ↓ | ↓ | ↓ |

For SCFAs s assay, groups which show no significance compared with naive group are represented by “-”, groups which significantly lower than naive group are represented by “↓”, groups which significantly higher than naive group are represented by “↑”.

**Supplementary Table 5.** Diversity and relative phylum abundance of gut microbiota associated with different pathogens treatment.

| **Group** | **Diversity and relative phylum abundance of gut microbiota** | | | | |
| --- | --- | --- | --- | --- | --- |
|  | **Chao-1**  **index** | **Shannon**  **index** | **Firmicutes** | **Bacteroidetes** | **Proteobacteria** |
| **Naive** | - | - | - | - | - |
| **Cj** | - | - | - | - | - |
| **T.g+Cj** | ↓ | ↓ | ↓ | - | ↑ |
| **T.g** | ↓ | ↓ | - | - | - |
| **S.t+Cj** | - | ↓ | - | - | ↑ |
| **S.t** | ↓ | ↓ | - | - | ↑ |
| **EIEC+Cj** | ↓ | ↓ | - | - | - |
| **EIEC** | ↓ | ↓ | ↓ | - | ↑ |
| **L.m+Cj** | ↓ | ↓ | - | - | - |
| **L.m** | ↓ | ↓ | - | - | ↑ |

For gut microbiota assay, groups which show no significance compared with naive group are represented by “-”, groups which significantly lower than naive group are represented by “↓”, groups which significantly higher than naive group are represented by “↑”.

**Supplementary Table 6.** Relative genus abundance of gut microbiota associated with different pathogens treatment.

| **Group** | **Relative genus abundance of gut microbiota** | | | | | | | | | |
| --- | --- | --- | --- | --- | --- | --- | --- | --- | --- | --- |
|  | **UEnt** | **UClo** | **ULach** | ***Cop*** | ***Dor*** | **US24** | ***Tur*** | ***Bac*** | ***Lact*** | ***Par*** |
| **Naive** | - | - | - | - | - | - | - | - | - | - |
| **Cj** | - | - | - | - | - | - | - | ↓ | ↑ | - |
| **T.g+Cj** | ↑ | ↓ | ↓ | - | - | - | - | - | ↓ | - |
| **T.g** | - | ↓ | ↓ | - | - | - | ↑ | - | ↑ | ↑ |
| **S.t+Cj** | ↑ | ↓ | ↓ | - | ↓ | - | - | - | - | - |
| **S.t** | ↑ | ↓ | ↓ | - | - | - | ↑ | - | ↑ | ↑ |
| **EIEC+Cj** | - | ↓ | ↓ | ↓ | - | - | ↑ | - | ↑ | - |
| **EIEC** | ↑ | ↓ | ↓ | - | ↓ | - | - | - | - | - |
| **L.m+Cj** | - | ↓ | ↓ | ↓ | - | - | - | - | - | ↑ |
| **L.m** | ↑ | ↓ | ↓ | - | - | - | - | - | ↑ | ↑ |

Relative abundance of genus unclassified Enterobacteriaceae (UEnt), unclassified Clostridiales (UClo), unclassified Lachnospiraceae (ULach), *Coprococcus* (*Cop*), *Dorea* (*Dor*), unclassified S24-7 (US24), *Turicibacter* (*Tur*), *Bacteroides* (*Bac*), *Lactobacillus* (*Lact*), *Parabacteroidesa* (*Par*) were shown. Groups which show no significance compared with naive group are represented by “-”, groups which significantly lower than naive group are represented by “↓”, groups which significantly higher than naive group are represented by “↑”.
